# Pretransplant HRCT Characteristics Are Associated with Worse Outcome of Lung Transplantation for Cystic Fibrosis Patients

**DOI:** 10.1371/journal.pone.0145597

**Published:** 2015-12-23

**Authors:** Gerdien Belle-van Meerkerk, Pim A. de Jong, Harold W. de Valk, Tim Neefjes, Frank A. Pameijer, Johanna M. Kwakkel-van Erp, Ed A. van de Graaf

**Affiliations:** 1 Department of Respiratory Medicine, University Medical Centre Utrecht, Utrecht, The Netherlands; 2 Department of Internal Medicine, University Medical Centre Utrecht, Utrecht, The Netherlands; 3 Department of Radiology, University Medical Centre Utrecht, Utrecht, The Netherlands; The Hospital for Sick Children and The University of Toronto, CANADA

## Abstract

**Objectives:**

Peri- and postoperative complications diminish the outcome of lung transplantation (LTx) in patients with cystic fibrosis (CF). We hypothesized that the degree of pathological findings on pre-LTx high resolution computed tomography (HRCT) is associated with higher morbidity and mortality in CF.

**Methods:**

All our CF patients undergoing LTx between 2001 and 2011 were included. HRCT examinations were evaluated according to a scoring system for pulmonary disease in CF patients, the Severe Advanced Lung Disease (SALD) score and for pleural involvement.

**Results:**

Fifty-three patients were included. Dominant infectious/inflammatory disease according to the SALD score was observed in 10 patients (19%). Five (50%) of those patients died within one week after LTx, compared to 2 (5%) patients without dominant infectious/inflammatory disease (p<0.001). This difference in survival percentage remained also significant in multivariate analysis. Patients with infectious/inflammatory disease received more packed red blood cells; 26 versus 8 in the first week (p<0.001). Pleural thickening was associated with higher requirement (10 units) for blood transfusion during LTx, compared to patients with normal pleura (4 units).

**Conclusions:**

The analysis of HRCT in CF patients according to the SALD score showed that dominant infectious/inflammatory disease is associated with a higher mortality after LTx. If confirmed in other studies, HRCT might aid estimation of surgical risk in some adult CF patients.

## Introduction

Lung transplantation is the final treatment modality for patients with end stage cystic fibrosis (CF) [1]. Peri- and postoperative complications are one of the major contributors to morbidity and mortality in CF patients after lung transplantation, especially in the first year [2,3]. To decrease early peri- and postoperative morbidity and mortality, it is important to identify clinical predictors for these complications, followed by elucidation of the causative mechanisms leading to them.

Few studies have addressed the question whether high-resolution computed tomography (HRCT) may help to predict perioperative complications [4,5]. As anatomical changes may play a major role in these events, for example pleural adhesion causing bleeding, it is likely that predictors can be found by imaging techniques. Loeve et al. described a method to study the extent of infectious/inflammatory lung damage in CF by analysis of HRCT [6,7]. With the severe advanced lung disease score (SALD), the total lung volume as visualized by HRCT is subdivided into four components; infection/inflammation, air trapping/hypoperfusion, normal/hyperperfusion and bulla/cysts. With this scoring system, better predictions of peri- and postoperative morbidity and mortality due to structural aberrations may become feasible.

Furthermore, these data may resolve a part of the mechanism of diabetes leading to a worse outcome after LTx [8,9]. It was described that the presence of diabetes is adversely related to survival after lung transplantation. In a CF cohort who underwent lung transplantation, mortality in diabetic patients was 44% whereas it was 6% in those without diabetes. Mortality was mainly due to hemorrhagic shock. The pathophysiological connection between diabetes and mortality after lung transplantation remains speculative, but the higher pulmonary infection rate in CF patients with diabetes, leading to more extensive destruction of pulmonary tissue, pleural adhesions and scarring, may play a role. We hypothesize that in patients with diabetes the severe advanced disease score is higher expressing more advanced structural disease in these patients leading to perioperative hemorrhage.

In the current study, we aimed to investigate whether the extent of structural lung disease, pleural disease and/or lymphadenopathy on HRCT is associated with peri- and postoperative lung transplantation morbidity and mortality.

## Materials and Methods

### Ethics statement

Data analyzed were collected data from clinical care, and all data were made anonymous and de-identified prior to analysis. No ethical approval is necessary in The Netherlands for this kind of research.

### Subjects

Patients who were evaluated for and underwent lung transplantation in our hospital between August 2001 and July 2011 were included in this study. Data were collected from clinical care. The pretransplant screening protocol included HRCT, diabetes screening, detailed patient history and physical examination. Patients with HRCT examinations that could not be retrieved were excluded from this study (n = 5).

### Lung transplantation protocol

Evaluation for LTx is performed if CF patients have one of the following criteria: a forced expiratory volume in one second (FEV_1_) less than 30% of predicted, PaO2 <7.3 kPa and/or PaCO2 >6.7 kPa, rapid clinical deterioration despite optimal treatment, frequent haemoptysis despite embolization, or therapy-resistant pneumothorax. During the screening process, possible contra indications are examined and data required for LTx are collected. Afterwards, the patient is added to the list awaiting LTx. When donor lungs are available, a double sided LTx is performed by clamshell thoracotomy. After LTx, patients are treated with standard immunosuppressive regime, consisting of basiliximab (induction), tacrolimus, mycophenolate mofetil and prednisone. In our center, the mean survival in CF patients thirty days, one year and five years after LTx are 83%, 74% and 69% respectively.

### High-resolution computed tomography

HRCT examinations were obtained in inspiration and expiration using a variety of CT scanners (single detector to 64-detector row from Philips Medical Systems, Best, The Netherlands). The HRCT examinations were scored by a single observer (Pim A. de Jong), for whom good reproducibility against other observers had been demonstrated previously [6]. The observer was blinded for the patients’ characteristics. The severe advanced lung disease score was used to assess the lungs as previous described by Loeve et al. [6]. Dominant infectious/inflammatory disease was arbitrarily defined as an infection/inflammation score ≥ 20% and an air trapping score <60% according to SALD. Additionally, subpleural adhesions (absent, mild, moderate, severe), pleural thickening (absent, mild, moderate, severe) and the largest lymph node (short axis diameter) at the left and right hilum and mediastinum were evaluated.

### Diabetes

Diabetes was diagnosed, according to the guidelines of the American Diabetes Association [10], when one of the following criteria was present: a) use of glucose lowering drugs, b) 2-h plasma glucose ≥ 11.1 mmol/L during a 75-gram oral glucose tolerance test (OGTT), or c) fasting glucose ≥ 7.0 mmol/L or random glucose ≥ 11.1 mmol/L on ≥ two separate days. Measurements of random blood glucose and, since 2008, annual 75 gram OGTT in non-diabetic subjects were performed. The diagnosis was only accepted when made in a period without infection (defined as an absence of antibiotics or CRP ≤ 10 mg/l), during stable use of corticosteroids.

### Microbiology

Sputum collected in the same period as the HRCT scan, was cultured and analyzed for Pseudomonas aeruginosa, Staphylococcus aureus, Aspergillus fumigatus and Burkholderia cepacia.

### Peri-/postoperative measurements

The number of packed red blood cells administrated and length of the surgical procedure were recorded. Parameters to assess peri- and postoperative bleeding were the total number of packed red blood cells in the first week (including the day of transplantation) and re-thoracotomy because of rebleeding. Mortality was assessed after one day, one week and thirty days.

### Statistical analysis

Continuous data are shown as the median with interquartile ranges because of skewed distribution; categorical data are shown as numbers with percentages. Differences between groups were compared with the non-parametric Mann-Whitney U test (continuous data) and a Chi-square or Fisher’s exact test (categorical data). For the analysis, subpleural adhesions and pleural thickening were grouped as absent/mild or moderate/severe. Dominant infectious/inflammatory disease was arbitrarily set as infection/inflammation score ≥ 20% and air trapping score <60%. The effect of dominant infectious/inflammatory disease on mortality was tested in multivariate analysis. Logistic regression was used to correct for six variables: age, body mass index, time on the waiting list, pretransplant diabetes, Pseudomonas aeruginosa in sputum culture and ischemic time of the donor lung. P-values <0.05 were considered statistically significant.

## Results

### Subjects

We included 53 CF patients in this study with a median age of 30 (IQR 23–39) years old, 32 of whom were male (60%). Diabetes was diagnosed before transplantation in 34 (64%) of the CF patients, of whom 27 patients (79%) already used insulin at screening. Patients underwent LTx after a median time on the waiting list of 13 (IQR 9–22) months. The median length of the surgical procedure was 6.5 hours (IQR: 6.0–8.0 hours) and 19 patients (36%) underwent a rethoracotomy because of rebleeding after transplantation. Total mortality after thirty days was 19% (n = 10). Three patients (6%) died in the first 24 hours post-transplant.

### HRCT and complications

HRCT examinations showed dominant infectious/inflammatory disease in 10 patients (19%). Comparisons between the groups with versus without dominant infectious/inflammatory disease are shown in Table 1. There was no difference between the groups in percentage of patients in whom Pseudomonas aeruginosa, Staphylococcus aureus and Aspergillus fumigates was cultured in their sputum. There was one patient with Burkholderia in each group. In the group without dominant infectious/inflammatory disease this patient had Burkholderia Cepacia in earlier sputum cultures and was regarded as colonized. Another patient in the group with dominant infectious/inflammatory disease had Burkholderia multivorans in the sputum culture. For patients with dominant infectious/inflammatory disease on HRCT, median duration of the surgical procedure was eight hours, compared to six hours for patients without dominant infectious/inflammatory disease (p = 0.005, Table 1). Patients with dominant infectious/inflammatory disease required significantly more packed red blood cells post-transplant (first week: 26 units of packed red blood cells versus 8 bags, p<0.001). Dominant infectious/inflammatory disease on HRCT was also associated with a higher mortality; five patients (50%) with dominant infectious/inflammatory disease died in the first week, compared to two patients (5%) in the group without dominant infectious/inflammatory disease on HRCT (p<0.001). Multivariate analysis on mortality after one week, with correction for age, body mass index, time on the waiting list, pretransplant diabetes, Pseudomonas aeruginosa and ischemic time of the lungs showed a persistent significant effect of the dominant infectious/inflammatory disease on mortality (p = 0.023).

**Table 1:** Baseline characteristics of 53 Cystic Fibrosis patients post-transplant.

Infectious/Inflammatory Disease	No dominance	Dominant	p-value
	N = 43 (81%)	N = 10 (19%)	
Age at LTx (years)	27 (23–39)	32 (28–38)	0.280
Gender (male)	27 (51%)	5 (50%)	0.456
Body Mass Index (kg/m2)	19.1 (18.4–22.6)	19.1 (17.8–21.8)	0.964
Time on waiting list (months)	12 (8–24)	15 (10–18)	0.856
*Diabetes*			
Pre-transplant diabetes (n)	25 (58%)	9 (90%)	0.058
Insulin at LTx (n)	22 (51%)	7 (70%)	0.281
*Microbiology*			
Pseudomonas Aeruginosa (n)	39 (91%)	8 (80%)	0.315
Staphylococcus Aureus (n)	4 (9%)	2 (20%)	0.315
Aspergillus Fumigatus (n)	7 (16%)	2 (20%)	0.778
Burkholderia Cepacia (n)^a^	1 (2.3%)	1 (10%)	0.345
*Peri- and postoperative measurements*			
Duration surgery (hours)	6 (5.5–7.5)	8 (6.5–11)	0.005
Packed red blood cells during LTx (n)	4 (2–8)	16 (7–26)	0.002
Packed red blood cells first week (n)	8 (2–13)	26 (11–71)	0.001
Rebleeding with rethoracotomy (n)	15 (35%)	4 (40%)	0.761
Death < 24 hours (n)	2 (5%)	1 (10%)	0.473
Death < 1 week (n)	2 (5%)	5 (50%)	<0.001
Death < 30 days (n)	5 (12%)	5 (50%)	0.005

^a^ One patient in No dominance-group had Burkholderia Cepacia in earlier sputum cultures and was regarded as colonized with Burkholderia Cepacia. One patient in Dominant group had Burkholderia Multivorans in the sputum culture.

An overview of the other structural changes found with HRCT is shown in Table 2. Moderate to severe pleural thickening on HRCT was associated with perioperative morbidity as measured by a longer duration of surgery and a higher number of packed red blood cells used (Table 2). Mediastinal and hilar lymph nodes of ≥ 15 mm and subpleural adhesions were not associated with peri-/postoperative morbidity and mortality.

**Table 2:** Association between high-resolution computed tomography findings and peri/post lung transplantation complications.

	N	Duration surgery (hours)	Packed cells during LTx (n)	Packed cells week 1 (n)	Rebleeding (n)	Death week 1 (n)	Death 30 days (n)
Dominant infectious/inflammatory disease							
Absent	43	6.0 (5.5–7.5)**	4 (2–8)**	8 (2–13)***	15 (35%)	2 (5%)***	5 (12%)**
Present	10	8.0 (6.5–11)	16 (7–26)	26 (11–71)	4 (40%)	5 (50%)	5 (50%)
Subpleural adhesions							
Absent/mild	33	6.0 (5.5–8.0)	4 (3–9)	8 (3–16)	10 (30%)	4 (12%)	5 (15%)
Mod./severe	20	7.0 (6.0–8.0)	7 (3–15)	12 (6–25)	9 (45%)	3 (15%)	5 (25%)
Pleural thickening							
Absent/mild	41	6.5 (5.5–8.0)*	4 (3–8,5)*	8 (3–15)*	13 (32%)	4 (10%)	6 (15%)
Mod./severe	11	8.0 (6.0–10)	10 (6–25)	26 (8–71)	6 (55%)	3 (27%)	4 (36%)
Hilar lymph nodes (right or left)							
<15mm	45	6.0 (5.5–8.0)	6 (3–10)	9 (4–21)	18 (40%)	6 (13%)	9 (20%)
≥15mm	8	7.5 (6.0–8.0)	4 (2–9)	8 (2–15)	1 (13%)	1 (13%)	1 (13%)
Mediastinal lymph nodes							
<15mm	37	6.0 (5.5–7.5)	6 (3–9)	8 (3–17)	11 (30%)	5 (14%)	7 (19%)
≥15mm	16	8.0 (6.0–8.0)	6 (2–16)	9 (3–23)	8 (50%)	2 (13%)	3 (19%)

* P<0.05,

** P<0.01,

*** P<0.001.

LTx = lung transplantation

### HRCT and diabetes

There was a definite trend towards more diabetic patients in the group with dominant infectious/inflammatory disease (n = 9 (90%)), compared to the group without dominant infectious/inflammatory disease (n = 25 (58%); p = 0.06, Table 3). Other structural changes on HRCT were not associated with diabetes before transplantation.

**Table 3:** Association between high-resolution computed tomography findings and diabetes before lung transplantation.

	N	Diabetes pretransplant (n)
Dominant infectious/inflammatory disease		
Absent	43	25 (58%)^#^
Present	10	9 (90%)
Subpleural adhesions		
Absent/mild	33	23 (70%)
Mod/severe	20	11(55%)
Pleural thickening		
Absent/mild	41	25 (61%)
Mod/severe	11	8 (73%)
Hilar lymph nodes (right or left)		
<15mm	45	30 (67%)
≥15mm	8	4 (50%)
Mediastinal lymph nodes		
<15mm	37	24 (65%)
≥15mm	16	10 (63%)

^#^ P = 0.058

## Discussion

This study demonstrated that more extensive structural changes associated with infectious/inflammatory disease according to the SALD score and pleural thickening on HRCT examinations at screening are associated with increased perioperative morbidity and postoperative mortality. We also found a trend for an association between infectious/inflammatory predominant disease and diabetes. HRCT may therefore be a helpful tool for estimating peri- and postoperative risk in lung transplant recipients with CF. Diabetes may play a role in the development of large airways disease and complications of lung transplantation, although this hypothesis requires further investigation.

The role of imaging in the pretransplant setting for CF patients is not adequately defined yet. Theoretically, pretransplant imaging may help to improve perioperative care and outcome. It is difficult to compare our current results with previous reports due to a different patient selection and the use of other outcome parameters. Marom et al. [11] included children and adults with CF and investigated the role of CT findings in addition to common clinical parameters to evaluate the usefulness of thoracic CT in CF patients before lung transplantation. It should be borne in mind that the results of studies in pediatric populations could not be translated automatically to patients who survived into adulthood without the need for lung transplantation at a younger age. Marom et al. concluded that there is no place for routine CT in CF patients before lung transplantation, because CT did not lead to changes in waiting list allocation and CT did not have an effect on surgical decision making during lung transplantation [11].

Apart from the investigated degree of pulmonary damage on pretransplant CT, pleural adhesions may be associated with hemorrhage during surgery and possible modern techniques may provide insight into the risk and outcome after LTx. Limited data are available on the relation between pleural disease in CF patients and outcome of lung transplantation. There have been two studies in pediatric populations presenting contradictory results. One study by Dosanjh et al. in 32 pediatric CF patients showed that 11 patients with severe pleural adhesions required a longer stay on the intensive care unit and a longer period of intubation than 13 patients with no or minimal pleural adhesions [5]. The other study, covering 35 CF patients and 11 patients requiring lung transplantation without CF, demonstrated that pleural scarring in pediatric CF patients was severe in just 4 patients (11%) without an effect on duration of surgery or number of blood transfusions [4]. Our study provides data limited to adult CF patients who were evaluated for lung transplantation. The structural changes seen on chest HRCT at screening are probably a result of chronic infection and inflammation during the life of the CF patient [12,13]. We showed that substantial and dominant HRCT patterns of infection/inflammation were associated with increased perioperative morbidity and postoperative mortality. Our 30-day mortality of 19% in this study is similar to earlier reports showing a 30-day mortality rate of 16–18% [14,15]. The infection/inflammation patterns in the lung may lead to lung tissue scarring and subsequently a higher surgical risk, measured by a longer duration and higher need for blood products as we showed in this study. Preoperative HRCT may thus be valuable for predicting the outcome of lung transplantation.

We also found a trend for an association between dominant infection/inflammatory disease on HRCT examinations and the presence of diabetes in CF patients. The fairly small sample population may be the explanation for why this association just failed to reach statistical significance. Pretransplant diabetes was diagnosed in 64% of our CF patients. In a general adult CF population a prevalence of 40–50% is given in literature [16,17]. However, a study by Hofer et al. on the same subpopulation of end-stage CF patients and using the same diagnostic criteria showed a prevalence of 65%, which is equal to our result [18]. Previous studies reported that CF patients with pre-existing diabetes have a poorer survival rate after lung transplantation than CF patients without pre-existing diabetes [8,19]. This study adds to the discussion as to whether diabetes increases lung transplant related mortality through pulmonary infections and a large number of infectious/inflammatory changes on HRCT. In addition to our own observations that patients with diabetes are more prone to develop infections, the latter was identified as a risk factor for treatment failure of pulmonary exacerbations in CF patients [20]. Although we cannot prove it with the current data, one can speculate that the increased structural lung changes and the higher mortality after LTx in CF patients with diabetes may be explained by a larger number of infections in these patients compared to CF patients without diabetes. Our results, although promising, are based on a single center and a fairly small population and further research is required to investigate if and how diabetes is causally associated with lung transplant complications.

The strengths of this study are a population consisting exclusively of adult patients, with a follow up according to protocol in a single center. We used a validated scoring system for CT investigation findings in CF patients and the diagnosis of diabetes was well defined.

There were also some limitations. We used the data from HRCT investigations at the time of evaluation of patients for lung transplantation. This means we do not have information on the development of new pulmonary pathology as visualized on HRCT during the period between screening and lung transplantation (median time on the waiting list was 13 months). In addition, before we analyzed the data, we arbitrarily defined predominant infection/inflammation as when a large part of the lung (≥20%) was involved without a major component of small airways disease (<60%). We thought this was a reasonable definition for infection/inflammation dominance, although cut-offs can always be debated and external validation is warranted. Finally, given our sample size our multivariate model concentrated on fairly common factors with a suspected moderate or severe effect on mortality. Rare or weaker factors could not be included in the model because of limited power.

In summary, our data show that the extent of infectious/inflammatory structural lung damage as visualized on HRCT is significantly related to higher peri- and postoperative morbidity and mortality. Furthermore, our study indicates that diabetes is associated with the predominant infection/inflammation pattern based on structural changes in the lungs. If confirmed in other studies, HRCT might aid estimation of surgical risk in some adult CF populations.

## Supporting Information

S1 Data (SAV)
